# Immunity to *Mycobacterium tuberculosis*

**DOI:** 10.1155/2011/406549

**Published:** 2011-07-10

**Authors:** James A. Triccas, Nathalie Winter, Carl G. Feng, Nicholas P. West

**Affiliations:** ^1^Department of Infectious Diseases and Immunology, University of Sydney, Sydney, NSW 2000, Australia; ^2^Infectiologie Animale et Santé Publique, INRA Centre de Tours, Nouzilly 37380, France; ^3^Laboratory of Parasitic Diseases, National Institute of Allergy and Infectious Diseases, National Institutes of Health, Bethesda, MD 20892, USA; ^4^Centenary Institute of Cancer Medicine and Cell Biology, Newtown, NSW 2042, Australia

Tuberculosis (TB) remains a major cause of mortality and morbidity worldwide. Currently, a third of the world's population is infected with *Mycobacterium tuberculosis*, the causative agent of TB, and annually there are 10 million new cases of clinical TB and approximately 2 million deaths [1]. TB kills more individuals each year than any other bacterial pathogen, and alarmingly, current control practices have not been able to significantly reduce the incidence of the disease over the past 15 years [1]. The current vaccine in use, *Mycobacterium bovis* bacille Calmette-Guérin (BCG), has been unable to limit the transmission of the disease, and the problem is compounded by the HIV/AIDS pandemic and the emergence of multidrug resistant strains of *M. tuberculosis* including extensively drug-resistant strains in multiple countries [2]. There is thus an urgent need to better understand the host response to *M. tuberculosis* and develop more effective strategies to control TB. 

This special issue contains original research reports and review articles covering a number of aspects of TB immunity. The first series of articles focuses on immune response to *M. tuberculosis* in humans. These studies range from the expression of cytolytic mediators following BCG vaccination in children (P. L. Semple et al.), control of antigen presentation function (A. Aquino et al.), changes in cellular makeup during postprimary TB (K. J. Welsh et al.), and treatment of disseminated infection in immuno-compromised individuals (A. A. Alangari et al.). The reviews of S. Meraviglia et al. and H. Saiga et al. describe the role of immune effectors in mycobacterial infection, while articles describing *M. tuberculosis* strain diversity (E. Nava-Agliluera et al.), TB in myelitis (Y. Feng et al.), and humans T- and B- cell responses to immunodominant mycobacterial antigens (G. C. Macedo et al.) also form part of this special issue.

 New insights on the interaction of *M. tuberculosis* with the host are also provided in this special issue. The reviews of M. Abebe et al., S. L. Sampson et al., and S. Ahmad describe pathogenic mechanisms and virulence factors expressed by *M. tuberculosis*, while the research articles of N. Sanarico et al. and E. Giacomini et al. investigate the transcriptional and cytokine response of host cells to *M. tuberculosis* infection. Reviews on TB transcriptomics (C. R. Zárate-Bladés et al.) and granuloma liquefaction (P.-J. Cardona) provide further insight into the disease process during *M. tuberculosis* infection. 

The development of new vaccines is a major goal of TB research programs, and this special issue contains a number of articles investigating vaccine design and testing in animal models. G. G. Guerrero and C. Locht report on the use of recombinant antigens to boost BCG-induced immunity, while C. Wang et al. and M. Okada et al. similarly investigate prime-boost approaches to develop more effective TB vaccine regimens. The immune response following vaccination with *M. tuberculosis* lipoproteins is described in the article of C. Palma et al., and the use of cattle as a model to study TB immunity is the focus of the review article of W. R. Waters et al. The special issue closes with an overview of biosensing technologies for detection of *M. tuberculosis* by Z. Zhou et al. 

In conclusion, the aspects of immunity to *M. tuberculosis* infection, host-pathogen interaction and vaccine development covered in this special issue may lead to future advances in the treatment and control of TB. 

*James A. Triccas* *Nathalie Winter* *Carl G. Feng* *Nicholas P. West*
